# Supplementary material for: Geographically Disperse, Culturable Seed-Associated Microbiota in Forage Plants of Alfalfa (Medicago sativa L.) and Pitch Clover (Bituminaria bituminosa L.): Characterization of Beneficial Inherited Strains as Plant Stress-Tolerance Enhancers
Source: Biology (Basel). 2022 Dec 16;11(12):1838. doi: 10.3390/biology11121838 (PMC9775229; doi:10.3390/biology11121838)
Supplement: Supplementary file 1 [file biology-11-01838-s001.zip › Supplementary Figures.pdf]

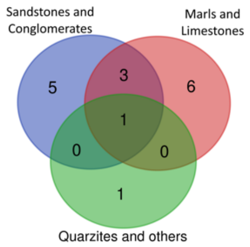

Names	Total	Elements
M+L/Quarzites/ S+C	1	*S. maltophilia*
M+L/S+C	3	*P. polymyxa, E. hormaechei, P. agglomerans*
S+C	5	*P. peoriae, P. xylanexedens, B. cereus P. megaterium, A. hermannii*
M+L	6	*B. nealsonii, P. amylolyticus, Erwinia sp. E. ludwigii, P. tundrae, P. pabuli*
Quarzites	1	*K. cowanii*

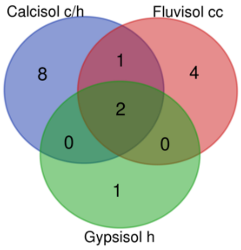

Names	Total	Elements
Calcisol c/h Fluvisol cc Gypsisol h	2	*S. maltophilia, P. polymyxa*
Calcisol c/h Fluvisol cc	1	*E. hormaechei*
Calcisol c/h	8	*P. xylanexedens, B. cereus, K. cowanii, Erwinia sp., E. ludwigii, P. tundrae, A. hermannii, P. pabuli*
Fluvisol cc	4	*P. peoriae, P. amylolyticus, P. megaterium, P. agglomerans*
Gypsisol h	1	*B. nealsonii*

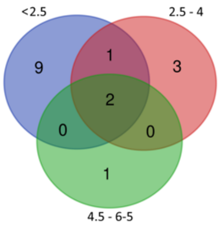

Names	Total	Elements
2.5 / 4.5 / 6.5	2	*S. maltophilia, P. polymyxa*
2.5 / 4.5	1	*E. hormaechei*
2.5	9	*P. xylanexedens, B. cereus P. amylolyticus, Erwinia sp. P. megaterium, E. ludwigii P. tundrae, P. pabuli, P. agglomerans*
4.5	3	*P. peoriae, B. nealsonii, A. hermannii*
6.5	1	*K. cowanii*

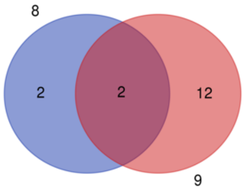

Names	Total	Elements
8 / 9	2	*S. maltophilia, P. polymyxa*
8	2	*P. peoriae, P. pabuli*
9	12	*P. xylanexedens, B. cereus, K. cowanii, B. nealsonii, P. amylolyticus, Erwinia sp., P. megaterium, E. ludwigii, P. tundrae, E. hormaechei, P. agglomerans, A. hermannii*

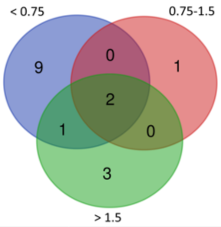

Names	Total	Elements
+1.5 / -0.75 / 0.75 to 1.5	2	*S. maltophilia, P. polymyxa*
+1.5 / -0.75	1	*E. hormaechei*
-0.75	9	*P. peoriae, B. nealsonii, K. cowanii, P. amylolyticus, Erwinia sp., P. tundrae, E. ludwigii, P. megaterium, P. agglomerans*
0.75 to 1.5	1	*A. hermannii*
+1.5	3	*P. xylanexedens, B. cereus, P. pabuli*

Supplementary Figure S1. Venn diagram for soil traits and bacteria species.

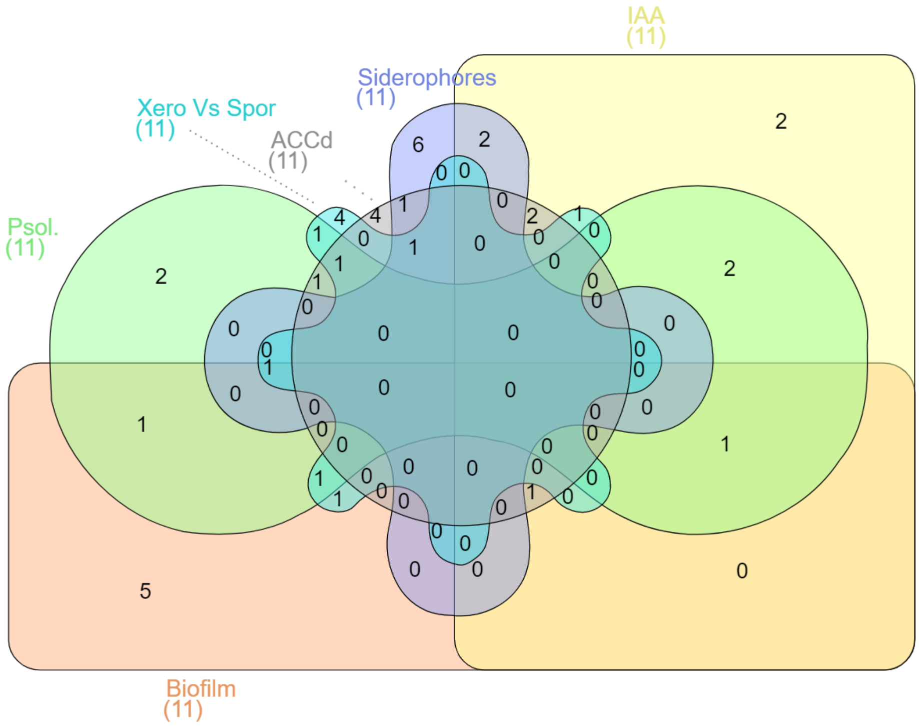

Supplementary Figure S2. Venn diagram for candidate selection criteria
