# Supplementary material for: Geographically Disperse, Culturable Seed-Associated Microbiota in Forage Plants of Alfalfa (Medicago sativa L.) and Pitch Clover (Bituminaria bituminosa L.): Characterization of Beneficial Inherited Strains as Plant Stress-Tolerance Enhancers
Source: Biology (Basel). 2022 Dec 16;11(12):1838. doi: 10.3390/biology11121838 (PMC9775229; doi:10.3390/biology11121838)
Supplement: Supplementary file 1 [file biology-11-01838-s001.zip › Supplementary Table S1.pdf]

Supplementary Table S1. Control strains for plant growth-promoting and drought resistance screening tests. 'Auxins' stand for auxins production; 'Biofilm', for biofilm structures production; 'ACCd', for the growth in ACC medium to determinate ACC deaminase production; 'P sol.', for phosphate solubilization; 'Sideroph.', for siderophores production; 'Sporul.', for sporulation; and 'Xerotol.', for xerotolerance. Values after '±' indicates standard deviation of recorded values.

Control strain	Test						
	Auxins	Biofilm	ACCd	P sol.	Sideroph.	Sporul.	Xerotol.
Escherichia coli MC4100	1.01 ± 0.05	0.22 ± 0.02	0.07 ± 0.01	(-)	(-)	(-)	(-)
Pseudomonas putida KT2440	25.11 ± 0.47	0.83 ± 0.11	0.44 ± 0.03	(+)	(+)	(-)	(-)
